# Methyl 9*H*-carbazole-9-acetate

**DOI:** 10.1107/S1600536809015141

**Published:** 2009-04-25

**Authors:** Yong-Jun He, Min-Hao Xie, Pei Zou, Ya-Ling Liu, Hong-Yong Wang

**Affiliations:** a Jiangsu Institute of Nuclear Medicine, Wuxi 214063, People’s Republic of China

## Abstract

The title compound, C_15_H_13_NO_2_, was synthesized by *N*-alkyl­ation of methyl bromo­acetate with 9*H*-carbazole. The carbazole ring system is essentially planar (mean atomic deviation = 0.0346 Å) and makes a dihedral angle of 86.5 (7)° with the methyl acetate group. Weak inter­molecular C—H⋯O hydrogen bonding is present in the crystal structure.

## Related literature

The title compound is an inter­mediate in the synthesis of -(9-carbazole) acetyl chloride, a novel fluorescence derivatization reagent, see: Xie *et al.* (2006 ▶); Bong *et al.* (1992 ▶). For bond distances, see: Allen *et al.* (1987 ▶). For the synthesis, see: Xie *et al.* (2006 ▶).
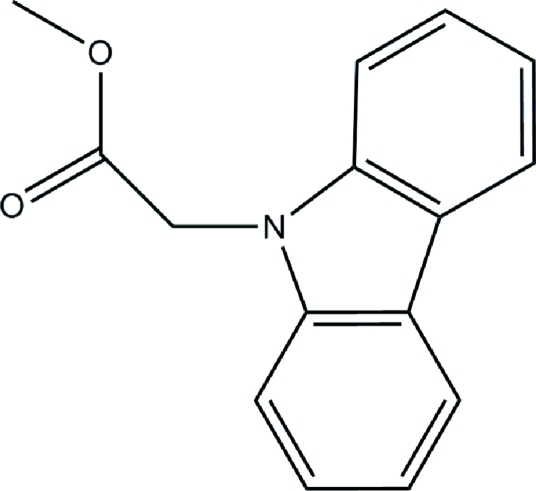


## Experimental

### 

#### Crystal data

C_15_H_13_NO_2_
*M* *_r_* = 239.26
Monoclinic, 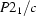

*a* = 10.875 (3) Å
*b* = 5.8773 (12) Å
*c* = 18.608 (4) Å
β = 103.599 (3)°
*V* = 1155.9 (5) Å^3^
*Z* = 4
Mo *K*α radiation
μ = 0.09 mm^−1^
*T* = 93 K
0.43 × 0.33 × 0.27 mm

#### Data collection

Rigaku SPIDER diffractometer
Absorption correction: none
8735 measured reflections
2615 independent reflections
1587 reflections with *I* > 2σ(*I*)
*R*_int_ = 0.041

#### Refinement

*R*[*F* ^2^ > 2σ(*F* ^2^)] = 0.055
*wR*(*F* ^2^) = 0.143
*S* = 1.00
2615 reflections
164 parameters
H-atom parameters constrained
Δρ_max_ = 0.33 e Å^−3^
Δρ_min_ = −0.36 e Å^−3^

### 

Data collection: *RAPID-AUTO* (Rigaku, 2004 ▶); cell refinement: *RAPID-AUTO*; data reduction: *RAPID-AUTO*; program(s) used to solve structure: *SHELXS97* (Sheldrick, 2008 ▶); program(s) used to refine structure: *SHELXL97* (Sheldrick, 2008 ▶); molecular graphics: *SHELXTL* (Sheldrick, 2008 ▶); software used to prepare material for publication: *SHELXTL*.

## Supplementary Material

Crystal structure: contains datablocks I, global. DOI: 10.1107/S1600536809015141/xu2513sup1.cif

Structure factors: contains datablocks I. DOI: 10.1107/S1600536809015141/xu2513Isup2.hkl

Additional supplementary materials:  crystallographic information; 3D view; checkCIF report

## Figures and Tables

**Table 1:** Hydrogen-bond geometry (Å, °)

*D*—H⋯*A*	*D*—H	H⋯*A*	*D*⋯*A*	*D*—H⋯*A*
C15—H15*B*⋯O2^i^	0.98	2.43	3.374 (3)	161

Symmetry code: (i) 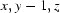.

**Fig. 1.**
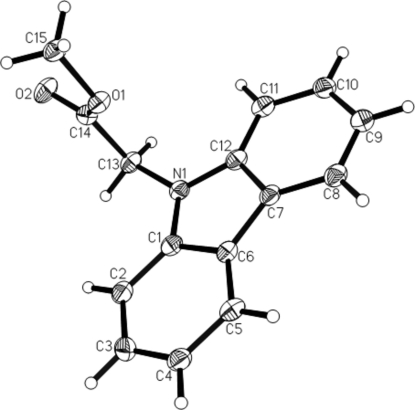
The molecular structure of the title compound with the atomic numbering scheme. Displacement ellipsoids were drawn at the 30% probability level.

## supplementary crystallographic information

### Comment

The title compound is useful as an intermediate in the synthesis of 2-(9-carbazole) acetyl chloride, a novel fluorescence derivatization reagent (Xie *et al.*, 2006; Bong *et al.*, 1992). We report here the crystal structure of (I), which is of interest to us in the field. The molecular structure of(I) is showed in Fig.1. The bond lengths and angles are within normal ranges (Allen *et al.*, 1987). The carbazole ring system is essentially planar with mean deviation of 0.0346 Å. The methylacetate substituent adopts a fully extended conformation, and its mean plane forms a dihedral angle of 93.5 (7)° with the carbazole mean plane. In the crystal structure weak C—H···O hydrogen bonding in present (Table 1).

### Experimental

The title compound was prepared by the method reported in literature (Xie *et al.*, 2006). The crystals were obtained by dissolving the title compound (0.1 g) in methanol (20 ml), and evaporating the solvent slowly at room temperature. Colorless prism-shaped crystals were formed after 3 d.

### Refinement

H atoms were placed in calculated positions and refined in ride mode with C—H = 0.95, 0.99 and 0.98 Å for aromatic, methylene and methyl H atoms. *U*_iso_(H) = 1.2*U*_eq_(C).

### Crystal data

**Table:**

C_15_H_13_NO_2_	*F*(000) = 504
*M**_r_* = 239.26	*D*_x_ = 1.375 Mg m^−^^3^
Monoclinic, *P*2_1_/*c*	Mo *K*α radiation, λ = 0.71073 Å
Hall symbol: -P 2ybc	Cell parameters from 3382 reflections
*a* = 10.875 (3) Å	θ = 3.3–27.5°
*b* = 5.8773 (12) Å	µ = 0.09 mm^−^^1^
*c* = 18.608 (4) Å	*T* = 93 K
β = 103.599 (3)°	Prism, colorless
*V* = 1155.9 (5) Å^3^	0.43 × 0.33 × 0.27 mm
*Z* = 4	

### Data collection

**Table:**

Rigaku SPIDER diffractometer	1587 reflections with *I* > 2σ(*I*)
Radiation source: Rotating Anode	*R*_int_ = 0.041
graphite	θ_max_ = 27.5°, θ_min_ = 3.3°
ω scans	*h* = −14→14
8735 measured reflections	*k* = −7→7
2615 independent reflections	*l* = −24→20

### Refinement

**Table:**

Refinement on *F*^2^	Primary atom site location: structure-invariant direct methods
Least-squares matrix: full	Secondary atom site location: difference Fourier map
*R*[*F*^2^ > 2σ(*F*^2^)] = 0.055	Hydrogen site location: inferred from neighbouring sites
*wR*(*F*^2^) = 0.143	H-atom parameters constrained
*S* = 1.00	*w* = 1/[σ^2^(*F*_o_^2^) + (0.0306*P*)^2^ + 1.86*P*] where *P* = (*F*_o_^2^ + 2*F*_c_^2^)/3
2615 reflections	(Δ/σ)_max_ < 0.001
164 parameters	Δρ_max_ = 0.33 e Å^−^^3^
0 restraints	Δρ_min_ = −0.36 e Å^−^^3^

### Special details

**Table:**

Geometry. All e.s.d.'s (except the e.s.d. in the dihedral angle between two l.s. planes) are estimated using the full covariance matrix. The cell e.s.d.'s are taken into account individually in the estimation of e.s.d.'s in distances, angles and torsion angles; correlations between e.s.d.'s in cell parameters are only used when they are defined by crystal symmetry. An approximate (isotropic) treatment of cell e.s.d.'s is used for estimating e.s.d.'s involving l.s. planes.
Refinement. Refinement of *F*^2^ against ALL reflections. The weighted *R*-factor *wR* and goodness of fit *S* are based on *F*^2^, conventional *R*-factors *R* are based on *F*, with *F* set to zero for negative *F*^2^. The threshold expression of *F*^2^ > σ(*F*^2^) is used only for calculating *R*-factors(gt) *etc*. and is not relevant to the choice of reflections for refinement. *R*-factors based on *F*^2^ are statistically about twice as large as those based on *F*, and *R*- factors based on ALL data will be even larger.

### Fractional atomic coordinates and isotropic or equivalent isotropic displacement parameters (Å^2^)

**Table:**

	*x*	*y*	*z*	*U*_iso_*/*U*_eq_	
O1	0.15039 (15)	0.6886 (3)	0.49433 (9)	0.0250 (4)	
O2	0.11310 (18)	1.0517 (3)	0.45700 (10)	0.0349 (5)	
N1	0.31648 (18)	0.7955 (3)	0.62011 (11)	0.0239 (5)	
C1	0.4231 (2)	0.6749 (4)	0.61296 (13)	0.0242 (5)	
C2	0.5141 (2)	0.7323 (4)	0.57434 (13)	0.0272 (6)	
H2	0.5081	0.8696	0.5468	0.033*	
C3	0.6132 (2)	0.5828 (5)	0.57759 (13)	0.0295 (6)	
H3	0.6767	0.6191	0.5520	0.035*	
C4	0.6228 (2)	0.3793 (5)	0.61751 (13)	0.0290 (6)	
H4	0.6912	0.2784	0.6179	0.035*	
C5	0.5332 (2)	0.3246 (4)	0.65647 (13)	0.0268 (6)	
H5	0.5400	0.1872	0.6840	0.032*	
C6	0.4324 (2)	0.4737 (4)	0.65483 (13)	0.0234 (5)	
C7	0.3267 (2)	0.4732 (4)	0.68959 (13)	0.0237 (5)	
C8	0.2899 (2)	0.3290 (4)	0.74042 (13)	0.0276 (6)	
H8	0.3364	0.1942	0.7568	0.033*	
C9	0.1848 (2)	0.3858 (5)	0.76644 (14)	0.0309 (6)	
H9	0.1602	0.2914	0.8021	0.037*	
C10	0.1143 (2)	0.5807 (5)	0.74077 (14)	0.0304 (6)	
H10	0.0412	0.6141	0.7585	0.036*	
C11	0.1483 (2)	0.7260 (5)	0.69037 (14)	0.0289 (6)	
H11	0.0994	0.8574	0.6730	0.035*	
C12	0.2565 (2)	0.6735 (4)	0.66592 (13)	0.0247 (5)	
C13	0.2590 (2)	0.9803 (4)	0.57265 (13)	0.0263 (6)	
H13A	0.3273	1.0713	0.5597	0.032*	
H13B	0.2146	1.0802	0.6012	0.032*	
C14	0.1662 (2)	0.9128 (4)	0.50173 (13)	0.0230 (5)	
C15	0.0599 (2)	0.6135 (4)	0.42803 (13)	0.0284 (6)	
H15A	−0.0252	0.6634	0.4301	0.034*	
H15B	0.0617	0.4471	0.4250	0.034*	
H15C	0.0821	0.6794	0.3844	0.034*	

### Atomic displacement parameters (Å^2^)

**Table:**

	*U*^11^	*U*^22^	*U*^33^	*U*^12^	*U*^13^	*U*^23^
O1	0.0257 (9)	0.0222 (9)	0.0240 (9)	−0.0003 (7)	−0.0001 (7)	0.0000 (7)
O2	0.0394 (11)	0.0295 (10)	0.0297 (10)	0.0044 (8)	−0.0043 (8)	0.0064 (8)
N1	0.0227 (10)	0.0239 (11)	0.0238 (10)	0.0030 (8)	0.0031 (8)	0.0028 (9)
C1	0.0241 (12)	0.0244 (13)	0.0217 (12)	0.0004 (10)	0.0005 (9)	−0.0026 (10)
C2	0.0275 (13)	0.0284 (13)	0.0236 (12)	−0.0001 (11)	0.0021 (10)	0.0023 (10)
C3	0.0298 (13)	0.0358 (15)	0.0233 (12)	−0.0010 (11)	0.0073 (10)	−0.0008 (11)
C4	0.0246 (12)	0.0358 (14)	0.0244 (12)	0.0034 (11)	0.0014 (10)	−0.0012 (11)
C5	0.0253 (12)	0.0237 (13)	0.0279 (13)	0.0033 (10)	−0.0006 (10)	0.0003 (11)
C6	0.0232 (12)	0.0247 (12)	0.0204 (11)	0.0005 (10)	0.0012 (9)	0.0003 (10)
C7	0.0226 (12)	0.0251 (12)	0.0204 (11)	−0.0018 (10)	−0.0007 (9)	0.0004 (10)
C8	0.0273 (13)	0.0290 (13)	0.0237 (12)	−0.0021 (11)	0.0001 (10)	−0.0008 (11)
C9	0.0271 (13)	0.0361 (15)	0.0278 (13)	−0.0034 (12)	0.0031 (10)	0.0010 (12)
C10	0.0246 (12)	0.0402 (16)	0.0258 (13)	−0.0006 (11)	0.0049 (10)	−0.0023 (12)
C11	0.0266 (13)	0.0310 (14)	0.0268 (13)	0.0031 (11)	0.0016 (10)	−0.0032 (11)
C12	0.0244 (12)	0.0244 (13)	0.0226 (12)	−0.0010 (10)	0.0000 (10)	−0.0024 (10)
C13	0.0281 (13)	0.0227 (13)	0.0250 (12)	0.0032 (10)	0.0001 (10)	0.0020 (10)
C14	0.0234 (12)	0.0191 (12)	0.0266 (12)	0.0016 (10)	0.0059 (10)	−0.0003 (10)
C15	0.0258 (12)	0.0310 (13)	0.0249 (12)	−0.0038 (11)	−0.0010 (10)	−0.0034 (11)

### Geometric parameters (Å, °)

**Table:**

O1—C14	1.332 (3)	C7—C8	1.397 (3)
O1—C15	1.454 (3)	C7—C12	1.416 (3)
O2—C14	1.211 (3)	C8—C9	1.383 (3)
N1—C12	1.388 (3)	C8—H8	0.9500
N1—C1	1.392 (3)	C9—C10	1.399 (4)
N1—C13	1.446 (3)	C9—H9	0.9500
C1—C2	1.394 (3)	C10—C11	1.381 (4)
C1—C6	1.407 (3)	C10—H10	0.9500
C2—C3	1.381 (4)	C11—C12	1.393 (3)
C2—H2	0.9500	C11—H11	0.9500
C3—C4	1.398 (4)	C13—C14	1.514 (3)
C3—H3	0.9500	C13—H13A	0.9900
C4—C5	1.383 (3)	C13—H13B	0.9900
C4—H4	0.9500	C15—H15A	0.9800
C5—C6	1.398 (3)	C15—H15B	0.9800
C5—H5	0.9500	C15—H15C	0.9800
C6—C7	1.445 (3)		
			
C14—O1—C15	115.67 (18)	C8—C9—C10	120.7 (2)
C12—N1—C1	108.52 (19)	C8—C9—H9	119.7
C12—N1—C13	124.4 (2)	C10—C9—H9	119.7
C1—N1—C13	125.0 (2)	C11—C10—C9	121.7 (2)
N1—C1—C2	129.4 (2)	C11—C10—H10	119.1
N1—C1—C6	109.1 (2)	C9—C10—H10	119.1
C2—C1—C6	121.5 (2)	C10—C11—C12	117.8 (2)
C3—C2—C1	117.7 (2)	C10—C11—H11	121.1
C3—C2—H2	121.2	C12—C11—H11	121.1
C1—C2—H2	121.2	N1—C12—C11	129.9 (2)
C2—C3—C4	121.8 (2)	N1—C12—C7	108.9 (2)
C2—C3—H3	119.1	C11—C12—C7	121.2 (2)
C4—C3—H3	119.1	N1—C13—C14	116.1 (2)
C5—C4—C3	120.2 (2)	N1—C13—H13A	108.3
C5—C4—H4	119.9	C14—C13—H13A	108.3
C3—C4—H4	119.9	N1—C13—H13B	108.3
C4—C5—C6	119.3 (2)	C14—C13—H13B	108.3
C4—C5—H5	120.4	H13A—C13—H13B	107.4
C6—C5—H5	120.4	O2—C14—O1	124.5 (2)
C5—C6—C1	119.5 (2)	O2—C14—C13	122.4 (2)
C5—C6—C7	133.7 (2)	O1—C14—C13	113.1 (2)
C1—C6—C7	106.8 (2)	O1—C15—H15A	109.5
C8—C7—C12	119.8 (2)	O1—C15—H15B	109.5
C8—C7—C6	133.5 (2)	H15A—C15—H15B	109.5
C12—C7—C6	106.6 (2)	O1—C15—H15C	109.5
C9—C8—C7	118.8 (2)	H15A—C15—H15C	109.5
C9—C8—H8	120.6	H15B—C15—H15C	109.5
C7—C8—H8	120.6		
			
C12—N1—C1—C2	−178.1 (2)	C6—C7—C8—C9	176.1 (3)
C13—N1—C1—C2	17.5 (4)	C7—C8—C9—C10	1.8 (4)
C12—N1—C1—C6	−0.3 (3)	C8—C9—C10—C11	−1.6 (4)
C13—N1—C1—C6	−164.7 (2)	C9—C10—C11—C12	−0.5 (4)
N1—C1—C2—C3	178.6 (2)	C1—N1—C12—C11	178.8 (2)
C6—C1—C2—C3	1.0 (4)	C13—N1—C12—C11	−16.7 (4)
C1—C2—C3—C4	0.5 (4)	C1—N1—C12—C7	1.0 (3)
C2—C3—C4—C5	−1.3 (4)	C13—N1—C12—C7	165.5 (2)
C3—C4—C5—C6	0.6 (4)	C10—C11—C12—N1	−175.1 (2)
C4—C5—C6—C1	0.8 (4)	C10—C11—C12—C7	2.5 (4)
C4—C5—C6—C7	−178.1 (2)	C8—C7—C12—N1	175.7 (2)
N1—C1—C6—C5	−179.7 (2)	C6—C7—C12—N1	−1.2 (3)
C2—C1—C6—C5	−1.6 (4)	C8—C7—C12—C11	−2.3 (3)
N1—C1—C6—C7	−0.5 (3)	C6—C7—C12—C11	−179.3 (2)
C2—C1—C6—C7	177.6 (2)	C12—N1—C13—C14	−77.4 (3)
C5—C6—C7—C8	3.7 (5)	C1—N1—C13—C14	84.6 (3)
C1—C6—C7—C8	−175.3 (3)	C15—O1—C14—O2	−1.6 (4)
C5—C6—C7—C12	−180.0 (3)	C15—O1—C14—C13	178.6 (2)
C1—C6—C7—C12	1.0 (3)	N1—C13—C14—O2	−179.0 (2)
C12—C7—C8—C9	0.2 (3)	N1—C13—C14—O1	0.8 (3)

### Hydrogen-bond geometry (Å, °)

**Table:**

*D*—H···*A*	*D*—H	H···*A*	*D*···*A*	*D*—H···*A*
C15—H15B···O2^i^	0.98	2.43	3.374 (3)	161

Symmetry codes: (i) *x*, *y*−1, *z*.
